# A simple vector system to improve performance and utilisation of recombinant antibodies

**DOI:** 10.1186/1472-6750-6-46

**Published:** 2006-12-07

**Authors:** Cecile D Martin, Gertrudis Rojas, Joanne N Mitchell, Karen J Vincent, Jiahua Wu, John McCafferty, Darren J Schofield

**Affiliations:** 1 Atlas of Protein Expression, Wellcome Trust Sanger Institute, Genome Campus, Morgan Building, Hinxton, Cambridgeshire, CB10 1HH, UK; 2 Present address: GlaxoSmithKline Medicines Research Centre, Biopharm CEDD-Cellular Immunology, Stevenage, UK; 3 Present address: Center for Genetic Engineering and Biotechnology, La Habana, Cuba; 4 Present address: Novartis Institutes for BioMedical Research (NIBR), Discovery Technologies, Basel, Switzerland

## Abstract

**Background:**

Isolation of recombinant antibody fragments from antibody libraries is well established using technologies such as phage display. Phage display vectors are ideal for efficient display of antibody fragments on the surface of bacteriophage particles. However, they are often inefficient for expression of soluble antibody fragments, and sub-cloning of selected antibody populations into dedicated soluble antibody fragment expression vectors can enhance expression.

**Results:**

We have developed a simple vector system for expression, dimerisation and detection of recombinant antibody fragments in the form of single chain Fvs (scFvs). Expression is driven by the T7 RNA polymerase promoter in conjunction with the inducible lysogen strain BL21 (DE3). The system is compatible with a simple auto-induction culture system for scFv production. As an alternative to periplasmic expression, expression directly in the cytoplasm of a mutant strain with a more oxidising cytoplasmic environment (Origami 2™ (DE3)) was investigated and found to be inferior to periplasmic expression in BL21 (DE3) cells. The effect on yield and binding activity of fusing scFvs to the N terminus of maltose binding protein (a solubility enhancing partner), bacterial alkaline phosphatase (a naturally dimeric enzymatic reporter molecule), or the addition of a free C-terminal cysteine was determined. Fusion of scFvs to the N-terminus of maltose binding protein increased scFv yield but binding activity of the scFv was compromised. In contrast, fusion to the N-terminus of bacterial alkaline phosphatase led to an improved performance. Alkaline phosphatase provides a convenient tag allowing direct enzymatic detection of scFv fusions within crude extracts without the need for secondary reagents. Alkaline phosphatase also drives dimerisation of the scFv leading to an improvement in performance compared to monovalent constructs. This is illustrated by ELISA, western blot and immunohistochemistry.

**Conclusion:**

Nine scFv expression vectors have been generated and tested. Three vectors showed utility for expression of functional scFv fragments. One vector, pSANG14-3F, produces scFv-alkaline phosphatase fusion molecules which offers a simple, convenient and sensitive way of determining the reactivity of recombinant antibody fragments in a variety of common assay systems.

## Background

The isolation of recombinant antibody fragments with unique binding specificities can be readily accomplished using antibody display methods such as phage display or ribosome display. In such display methods large naïve libraries are generated where the gene encoding an antibody is physically linked to the resulting antibody protein (in the form of a single chain Fv fragment (scFv)). The binding properties of the antibody fragment are then used to isolate the encoding gene [1]. While phage display vectors are designed to display recombinant antibodies on the surface of phage, they are not necessarily optimal for scFv expression and often selected antibody genes are sub-cloned into a dedicated soluble antibody fragment expression vector. Sub-cloning into an expression vector is also a requirement with ribosome display. The aim of the work detailed in this paper is to improve the production and utilisation of recombinant scFvs and generate a panel of expression vectors to facilitate this. The backbone expression vector utilises the high level expression promoter T7*lac*, driving expression of scFv to the bacterial periplasmic space via a *pelB *signal peptide sequence. Resultant products are fused with a six histidine tag for one step immobilised metal affinity chromatography (IMAC) and a tri-FLAG epitope tag for detection.

scFv expression was performed in two *Escherichia coli *strains for comparison. For standard periplasmic expression of the scFvs and their derivatives, the BL21 (DE3) strain was used. This strain carries a chromosomal copy of T7 RNA polymerase, under the control of the *lac*UV5 promoter. This can be induced using IPTG or a simple auto-induction medium as we show here. T7 RNA polymerase can be induced resulting in expression of genes driven from the T7*lac *promoter. This strain is also deficient in both *lon *and *ompT *proteases. We also investigated expression of scFvs directly into the cytoplasm using a vector which lacked a *pelB *signal peptide sequence. This was carried out in an *E. coli *K12 derivative strain [2], which has mutations knocking out both thioredoxin reductase (*trx*B) and glutathione reductase (*gor*) creating a more oxidizing cytoplasmic environment to facilitate disulphide bond formation.

In this study we compare the expression levels and ELISA signals from crude bacterial cell extracts of a number of C-terminal modifications to the standard scFv format, including a free C-terminal cysteine residue [3,4], and maltose binding protein (MBP). MBP has previously been shown to act as a solubility enhancing tag for recombinant proteins [5]. Antibody fragments were also fused to a variant alkaline phosphatase gene with enhanced catalytic activity encoded by a D153G and D330N mutation [6,7]. The alkaline phosphatase fusion drives scFv dimerisation and provides a simple enzymatic fusion partner to facilitate direct detection of scFv fusions [6,8-10]. We illustrate the benefit of this system in a number of common assays including ELISA, western blot and immunohistochemistry.

## Results

### Vector construction

**Figure 1:**
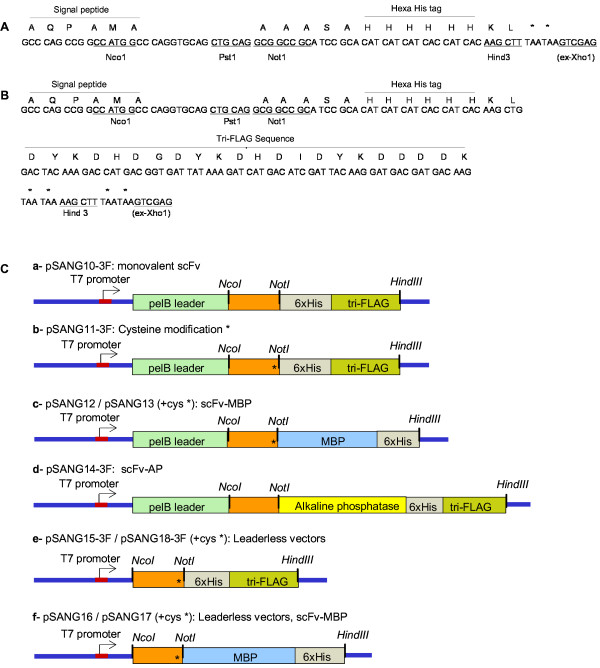
**Construction of pSANG vectors**. **A.** Sequence of the basic vector pSANG-10, which was created by inserting an oligo between a *Bgl*I site (GCCnnnnnGGC) and *Xho*I site (CTCGAG now destroyed) downstream of the promoter within the vector backbone derived from pET26 (+) plasmid (Novagen, San Diego, CA). The inserted sequence cloned between these restriction sites is shown. Translated sequences from the end of the signal peptide and hexa-histidine tag are indicated. The scFv encoding gene is sub-cloned at the *Nco*I*/Not*I site and the *Hind*III site is available for insertion of peptide tags and fusion partners. **B. **For comparison the sequence of pSANG10-3F is shown where a tri-FLAG tag has been inserted. An oligonucleotide encoding the tri-FLAG sequence was cloned into the *Hind*III site of pSANG10 destroying the site at 5' end and retaining it at 3' end for future use. **C. **Schematic view of the expression cassettes of the pSANG vectors. **a-d**: vectors used with BL21 (DE3) host strain for periplasmic expression. **e-f**: vectors used with Origami 2™ (DE3) host strain for cytoplasmic expression. MBP= Maltose Binding Protein; * = serine replaced by a cysteine.

Figure 1 shows a schematic representation of the expression cassette for each vector, and the pertinent properties of each of the vectors are detailed in Table 1. All vectors are driven by a T7 promoter and contain a hexa-histidine tag for purification of scFv. The original vectors were created with a *Hind*III site for tag cloning (e.g. pSANG-10, Figure 1a) and, in the bulk of the work described here, a tri-FLAG tag has been introduced at this site (e.g. pSANG10-3F, Figure 1b). Figure 1c shows alternative constructs created from this basic backbone. Essentially there were five different scFv expression forms: scFv only (scFv), scFv plus a C-terminal cysteine residue (scFv-cys), scFv fused to maltose binding protein (scFv-MBP), scFv-MBP plus a C-terminal cysteine residue (scFv-MBP-cys), and scFv fused to bacterial alkaline phosphatase (scFv-AP). Additional constructs without the *pelB *leader sequence (leaderless) were made in order to produce the scFv, scFv-cys, scFv-MBP, and scFv-MBP-cys forms expressed in the cytoplasm rather than the periplasmic space in Origami 2™ (DE3) cells. The Origami 2™ (DE3) strain is a *gor/trx*B mutant which can facilitate disulphide bond formation by creating an oxidising cytoplasmic environment.

### scFv expression induced by auto-induction medium is equivalent to IPTG induction

Use of the T7 promoter in the pSANG vectors requires the use of DE3 lysogen strains such as BL21 (DE3), where there is an integrated copy of T7 RNA polymerase. Expression is controlled by the *lac *promoter driving expression of T7 RNA polymerase which in turn acts on the T7 promoter to induce scFv expression. The *lac *promoter is normally induced by the addition of IPTG in mid-log phase growth. Studier [11] describes the development of a growth medium-controlled induction system, which does not require user intervention to switch on recombinant protein expression. The growth medium contains a controlled amount of carbon sources for the bacteria to utilize for growth, and expression is tightly controlled by catabolite repression. As the bacterial culture density increases the catabolite repression is diminished by loss of the carbon source. When this begins, expression from the *lac *promoter is turned on due to the presence of lactose in the induction medium.

**Figure 2:**
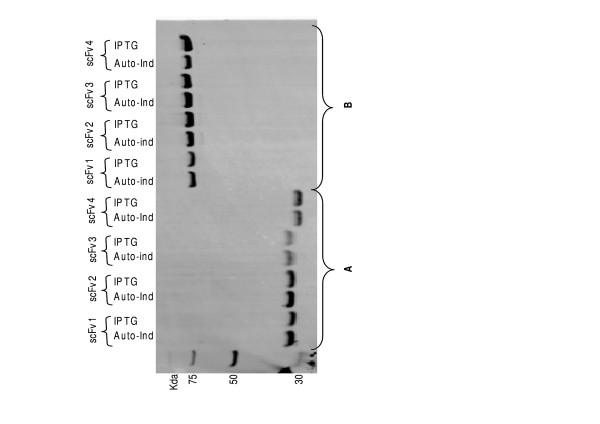
**A comparison of scFv expression using auto-induction media and IPTG induction**. **A**: ScFv expressed from pSANG10-3F; **B**: ScFv-AP expressed from pSANG14-3F. BL21 (DE3) were grown overnight at 30°C using either auto-induction media (Auto-Ind) or mid-log phase induction with IPTG. The bacterial cell pellet was lysed and analysed by western-blot (10 μl/lane) using an anti-histidine tag mouse monoclonal antibody and Cy5 labelled anti-mouse detection system.

We examined the expression levels achieved with four scFv clones expressed in both the scFv and scFv-AP formats using auto-induction medium and the more standard induction medium of 2xYT supplemented with IPTG. Figure 2 shows that the two systems give comparable expression levels. The advantage of the auto-induction medium is that optical density monitoring is not required prior to induction. The cultures are simply inoculated and then harvested 20 h after incubation at 30°C. For all subsequent experiments auto-induction medium was used for expression of the different scFv formats.

### Effect of fusion partner and site of expression on yield of scFv

Antibody fragment clones specific for human collagen type VI and human desmin were expressed in a variety of scFv formats in two different bacterial strains. The total and active yields of the antibody fragments were assessed by western blot and an antigen binding ELISA respectively. Each clone was grown overnight in auto-induction medium, the bacteria pelleted and a detergent lysis performed. The different scFv formats were run in SDS-PAGE gels, blotted onto membranes and probed with an anti-FLAG tag monoclonal antibody coupled to a fluorescent tertiary antibody to assess both the quality and quantity of the scFv produced. We have previously shown that fluorescent western blots can be used to quantitate protein production [5].

**Figure 3:**
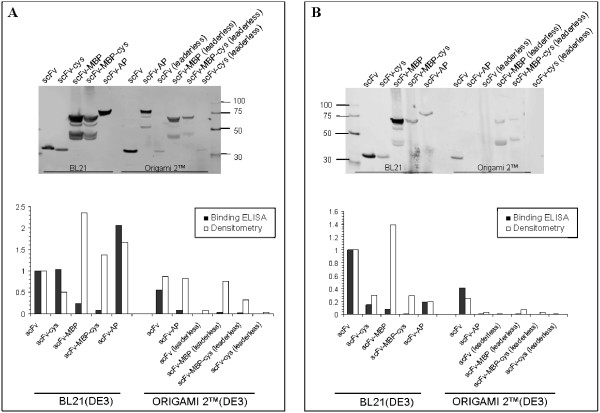
**Effect on yield of fusion partner and site of expression**. Cells were grown overnight at 30°C in auto-induction media [11]. The bacterial cell pellet was lysed, clarified and three-fold dilutions of each lysate were analysed by western-blot for the protein expression and by ELISA for scFv binding activity. Representative ELISA signals from the linear part of the titration curve (1:81 dilution) were plotted relative to those obtained for pSANG10-3F expressed scFv. **A**: scFv specific for collagen type VI; **B**: scFv specific for desmin (clone C10). A representative western blot showing total protein yield for each scFv format is shown in the upper part of panel **A **and **B**. The graphs in the lower part of **A **and **B **show normalised ELISA data and normalised densitometry values from the western blot MBP: Maltose Binding Protein. AP: Alkaline Phosphatase.

Densitometry was also used to quantitate the yield of each scFv format and the data were normalised to the values obtained for the standard scFv format expressed in BL21 (DE3) cells (summarised in Figure 3). Antigen binding ELISAs were also carried out to determine the relative amount of biochemically active recombinant protein in each preparation. Figure 3 panel A shows the results obtained for the collagen VI specific clone. In the ELISA, the scFv-Cys format had an equivalent activity to the standard scFv. ScFv-MBP fusion proteins were not as active in the ELISA as the standard scFv. The addition of a free C-terminal cysteine did not improve the activity of the scFv-MBP format in the ELISA. Western blot analysis revealed the reduced activity in ELISA was not due to diminished expression levels. In fact expression levels of both scFv-MBP fusions were far higher than the standard monovalent scFv suggesting interference in antigen binding with scFv-MBP fusions. There was also evidence of lower molecular weight bands which probably represent proteolytic cleavage products. The scFv-AP format produces scFv fused to bacterial alkaline phosphatase, which is enzymatically active only as a dimer. The ELISA activity for this scFv-AP clone was superior to the standard scFv and yields were also higher as determined by western blot. Figure 3B shows the results for an anti-desmin clone. The results for this antibody fragment clone are in broad agreement with those obtained for the anti-collagen VI clone. However, notably the yield and activity of the scFv-AP format was reduced compared to the standard scFv format for this clone. A third scFv clone specific for human HER2 was also tested in the panel of vectors and gave broadly similar results to those obtained above (data not shown).

All of the above clones were expressed in BL21 (DE3) cells and the polypeptides generated are directed to the periplasmic space of the bacteria by the presence of a signal peptide sequence. Two vectors, pSANG15-3F and pSANG18-3F, were designed without a signal peptide to allow expression of the scFv and scFv-cys formats in the cytoplasm. To facilitate disulphide bond formation, the redox modified bacterial strain Origami 2™ (DE3) was used. In order to benchmark these clones, we also expressed the corresponding standard periplasmic scFv and scFv-AP formats from the pSANG10-3F and pSANG14-3F vectors respectively in the Origami 2™ (DE3) strain. In conclusion, expression of all constructs in Origami 2™ cells was compromised relative to BL21 (DE3) cells and there was no evidence of improved expression using leaderless constructs (Fig 3A,3B) and these were dropped from further studies.

From this initial screen there are three useful scFv formats: scFv, scFv-cys, and scFv-AP derived from the expression vectors, pSANG10-3F, pSANG11-3F, and pSANG14-3F respectively.

### Direct detection of scFv fused to bacterial alkaline phosphatase

Fusion of the scFv to alkaline phosphatase allows direct detection of binding. ScFv-AP specific for collagen VI, desmin, and HER2 were grown overnight in auto-induction medium. After pelleting, and detergent lysis of the bacteria to release the scFv-AP, the clarified lysate was diluted in assay buffer and the scFv-AP clones tested directly for binding to their cognate antigens in ELISA by adding substrate. The fusion to alkaline phosphatase means this can be done directly without adding secondary reagents. This confirmed that the scFv-AP fusions work very well in a simple direct detection ELISA (not shown).

**Figure 4:**
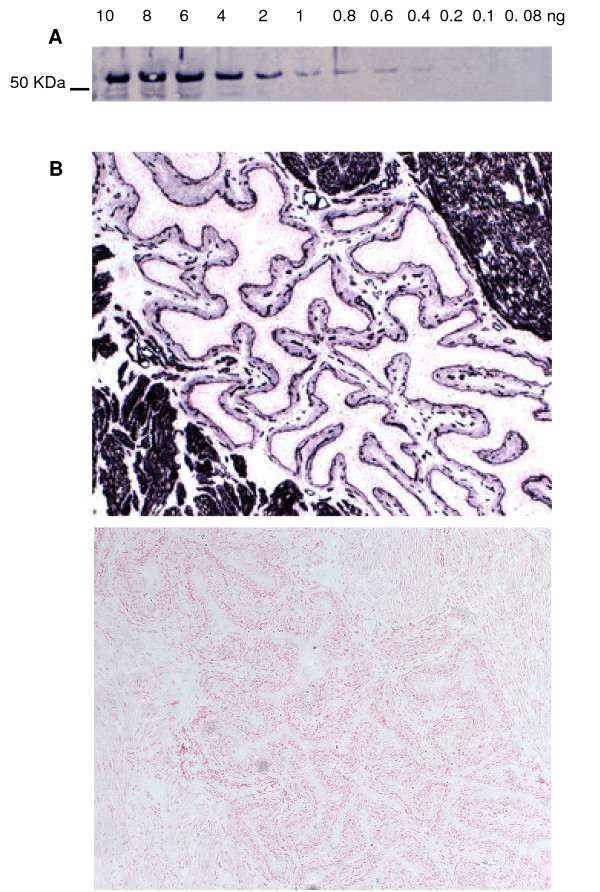
**Direct detection of scFv binding by using the alkaline phosphatase fusion partner**. **A.** Direct detection in western-blot using scFv-AP expressed from pSANG14-3F. Recombinant human desmin directly detected with scFv-AP from bacterial cell extract diluted 1:5 using NBT/BCIP. **B**: Direct detection of collagen VI in immunohistochemical staining of acetone-fixed frozen mouse bladder using 0.2 μg/ml scFv-AP detected by NBT/BCIP (upper panel). The lower panel shows the same tissue incubated with NBT/BCIP in the absence of an scFv-AP.

The scFv-AP format provides a convenient and easy to use option for probing western blots for the presence of target antigen. Figure 4A demonstrates the sensitivity of an scFv-AP clone directed to recombinant human desmin. Doubling dilutions of the antigen were run on an SDS-PAGE gel and transferred to a PVDF membrane, blocked and then probed with a single dilution of crude bacterial cell extract from an overnight induction. The assay was again developed without the addition of an anti-tag secondary antibody using a standard colorimetric alkaline phosphatase substrate, NBT/BCIP. The scFv-AP was able to detect the presence of antigen down to 0.4 ng/lane.

Figure 4B highlights the utility of the scFv-AP expression system in offering a simple direct detection system in IHC. Here strong staining of human collagen VI is demonstrated at 0.2 μg/ml of scFv-AP (Figure 4B upper panel), detecting the antibody fragment binding with the addition of NBT/BCIP colorimetric substrate whereas no staining is observed with substrate alone (Figure 4B lower panel). Thus these results demonstrate that direct detection of alkaline phosphatase fusions is possible in a variety of applications. It is our experience, however, that addition of an alkaline phosphatase conjugated secondary antibody can be used to increase sensitivity further by dual labelling with alkaline phosphatase (see below).

### Enhanced performance of scFv following dimerisation by alkaline phosphatase

A panel of five scFv clones recognising the same epitope of the breast cancer marker HER2 were used to investigate the effect of valency/affinity on performance. The affinities of this panel of five scFv clones have been determined previously [12-14], with a range of equilibrium dissociation constants (Kd) ranging from a lowest of 320 nM to the highest at 0.013 nM.

#### ELISA

**Figure 5:**
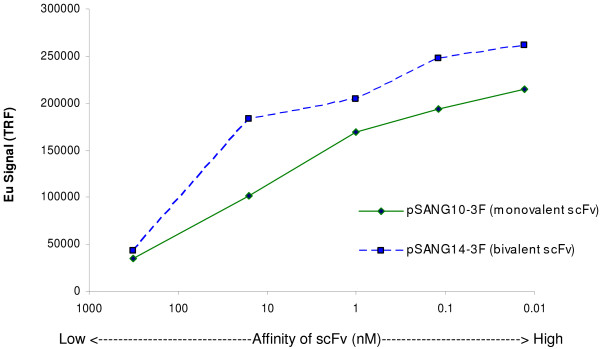
**Effect of alkaline phosphatase fusion on performance in ELISA**. A series of scFvs, as monomeric scFv or bivalent scFv-AP, recognising the same epitope of HER-2 with different affinities were tested at equimolar scFv concentration (1 × 10^-7 ^M) on ELISA wells coated with HER-2. Detection was by time resolved fluorescence using Europium-N1 labelled anti-FLAG M2 antibody.

Increasing the valency of the HER2 specific scFvs resulted in a greater sensitivity in an ELISA format. All five scFv clones were assayed as both monovalent scFv and bivalent scFv-AP at equimolar concentrations (1 × 10^-7 ^M) on HER2 coated wells. The monomeric molecular weight for the scFv-AP construct was used to calculate molarity. Thus any difference in relative signal is therefore caused by increased valency rather than quantitative effects of two detection tags on the bivalent scFv-AP format compared with one on the standard monovalent format. Binding was detected by time resolved fluorescence using a europium-labelled anti-FLAG secondary antibody. The results shown in Figure 5 clearly demonstrate that the clones expressed as bivalent scFv-AP exhibit an improved sensitivity in this assay format.

#### Immunohistochemistry

**Figure 6:**
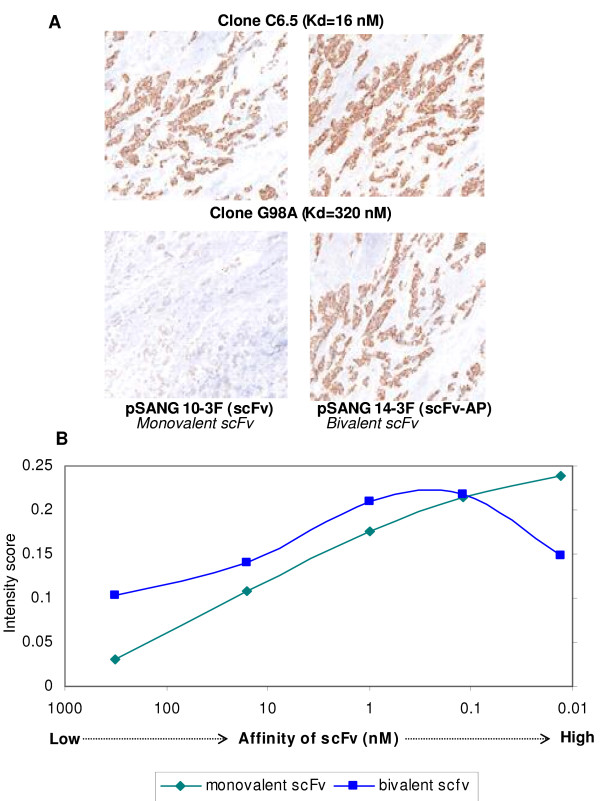
**Effect of alkaline phosphatase fusion on performance in IHC. A. **scFv-APs demonstrate improved sensitivity in IHC. HER2 clones are more sensitive when expressed in pSANG14-3F than pSANG10-3F Monovalent G98A, with an affinity of 320 nM, gave weakly positive staining of the antigen in breast cancer tissue. Whereas, bivalent G98A gave staining equivalent to the monomeric scFv clone C6.5, which has a 20-fold higher affinity. Bivalency does not visually improve C6.5 staining. Detection was by anti-FLAG antibody coupled to biotin with the addition of streptavidin-horse-radish peroxidase and a tyramine signal amplification step. **B. **Image analysis was used to quantify the staining intensities of all five scFvs as both monomeric scFv and bivalent scFv-AP.

The same HER2 clones were used to demonstrate the improved sensitivity of bivalent scFv-AP in immunohistochemical staining of acetone-fixed frozen human breast carcinoma sections. For example scFv clones, G98A and C6.5 have equilibrium dissociation constants of 320 and 16 nM respectively. The clones were assayed at identical protein concentrations and binding detected using anti-FLAG and a tyramine-DNP amplification system. The results shown in Figure 6A demonstrate that HER2 staining for the monovalent clone G98A is weak, although positive, and for the C6.5 clone, with a 20-fold improvement in affinity, staining of HER2 is strong. In contrast, staining of HER2 using G98A as an scFv-AP dimer is as strong as the monovalent C6.5 clone. The increased avidity for HER2 generated by making C6.5 bivalent has less effect probably due to a threshold of affinity required for good immunostaining by this antibody-epitope combination. Automated digital image analysis provides a means to quantify immuno-reactivity in tissue microarrays or tissue sections. The staining intensities for all five scFv clones were quantitated and are plotted in Figure 6B.

#### Western blot

**Figure 7:**
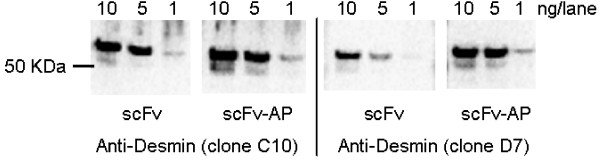
**Effect of alkaline phosphatase fusion on performance in Western blot**. scFv-APs demonstrate improved sensitivity in Western blot. Recombinant human desmin was detected with equimolar concentrations of anti-desmin scFv clone C10 (applied at a concentration of 2 × 10^-8 ^M) expressed as either scFv or scFv-AP formats. Detection was by anti-FLAG antibody coupled to horse-radish peroxidase and addition of ECL western-blotting substrate.

The bivalent scFv-AP format demonstrated superior sensitivity over the monovalent version in detecting desmin from a recombinant source in western blot. Using anti-desmin scFv clones as a model, we performed western blots with dilutions of recombinant human desmin (10, 5, and 1 ng/lane) and probed the blots with purified scFv and scFv-AP clones diluted, as previously, to equimolar concentrations (2 × 10^-8 ^M). Binding of the scFv clones was determined by addition of an anti-FLAG alkaline phosphatase conjugate (not shown) or an anti-FLAG-HRP conjugate using ECL western blotting substrate. For the two anti-desmin clones (Figure 7) the bivalent scFv-AP demonstrates superior sensitivity over the monovalent scFv by 2.5-fold and 7.6-fold for clone C10 and D7 respectively, as determined by densitometric analysis of the western blots. Detection of endogenous desmin in muscle lysates was also demonstrated (not shown).

## Discussion

Phage display and other methods of generating recombinant antibodies have proven to be a powerful technology for generating antibody specificities. The widespread adoption of this technology outside of core labs however has been limited for a number of reasons. This includes lack of access to clones of desired specificity and problems of variable levels of production where clones are available. Efforts are being initiated to generate and distribute recombinant clones for the research community *e.g. *the Atlas of Protein Expression [15], the Proteome Binders Consortium [16], and the National Cancer Insititute [17].

To help deal with the issue of production of recombinant proteins in non-core labs, we have devised a simple vector system to facilitate production of tagged products. The system described here utilises auto-induction medium which does not require user intervention to switch on recombinant protein expression. In addition all products have a hexahistidine tag for IMAC purification, with options for additional detection tags to be added e.g. tri-FLAG tag as described here.

One particularly useful construct fuses recombinant antibody fragments to an optimised variant of bacterial alkaline phosphatase, a dimeric, periplasmic enzyme. This allows a simple assessment of production of fusion protein in culture supernatants or lysates, without the need for secondary detection reagents, based on alkaline phosphatase activity by ELISA. We also demonstrate direct detection of binding based on alkaline phosphatase in commonly used assays such as immunohistochemistry and western blotting (although more sensitive results are achieved by indirect methods using secondary anti-FLAG tag reagents).

An additional benefit of this construct is that antibody dimerisation is driven by fusion to alkaline phosphatase. An alternative version was produced which includes a free C-terminal cysteine residue which also enables dimerisation (not shown) as well as facilitating the conjugation of a reporter molecule such as biotin or FITC [3]. Theoretically increasing valency has the benefit of increasing binding affinity of the dimeric molecule. We show this in practice by comparing alkaline phosphatase fused single chain Fvs (scFv-AP) with their unfused counterparts in ELISA, western and immunohistochemistry. This was done by indirect detection using the tri-FLAG tag present on all formats. The degree of improvement in sensitivity for the scFv-AP format over the standard scFv format for any given assay will be dependent on antibody affinity, assuming an equal fraction of active recombinant protein for any given preparation. This was highlighted using a panel of anti-HER2 scFvs with a range of affinities to the same epitope. We demonstrated that, when compared on a molar basis, the alkaline phosphatase fused scFvs were more active in ELISA. Furthermore, using immunohistochemistry, enhanced staining of HER2 positive breast cancer tissue samples was observed with alkaline phosphatase fused scFvs compared to the monomeric counterpart. However, the difference in staining diminished with increasing affinity of the monomeric scFv.

It should be noted, that the standard scFv format can spontaneously form dimers and higher order multimers. These multimers are not stable and the proportions of monomer, dimer, and higher order multimer after purification can change over time in storage. The ability of scFvs to form multimers again is a property of the individual clones and some are more susceptible than others to this phenomenon. The degree of multimerisation of the standard scFv may alter the relative performance of the scFv when compared to the scFv-AP format.

The performance of the different scFv formats was assessed in terms of total recombinant protein yield using a western blot of detergent lysed bacterial pellets to detect the presence of epitope tagged scFv protein. This gave a direct comparison of the yield of each of the scFv formats retained in the cell after production. As a total cell lysate however, it provides an estimate of the total recombinant protein produced, including not only the correctly folded active scFv format but also incorrectly folded, unfolded and aggregated recombinant protein. Hence the relative amount of active scFv format in each preparation was also estimated by measuring the fraction of scFv that was active in binding to antigen coated ELISA wells. Together the two results clearly indicate the relative levels of expressed to active recombinant protein. Although yields vary on a clone by clone basis, in general the specific activity is relatively constant with the exception of fusions to maltose binding protein. With MBP, the yield of scFv protein increased while the binding activity reduced (*e.g. *Fig 3). The improvement in total protein may be due to MBP acting to form soluble inclusion bodies [18], which in turn may limit access of the scFv to antigen.

It is known that leakage of scFv into the culture medium occurs and this is dependent on the type of growth medium used for expression. Leakage can even be enhanced by the addition of sucrose to the growth medium [19]. It is our experience that the leakage of the larger scFv fusion proteins (*e.g. *scFv-AP) is reduced compared to the standard scFv format. By choosing to compare only the recombinant scFv protein retained within the bacterial cell therefore we may have underestimated the total production, particularly for the standard scFv. While measurement and recovery of scFv from culture medium is achievable, it is not a straight forward one-step Ni-NTA purification procedure [19]. Thus we have deliberately chosen to work with the periplasmic fraction to facilitate concentration and purification of resulting product.

## Conclusion

We have investigated a number of different scFv formats for expression and have generated three simple expression vectors (pSANG10-3F, pSANG11-3F and pSANG14-3F) for the production of soluble recombinant scFv fragments. These vectors allow rapid cloning of whole populations of scFv fragments selected from phage display antibody libraries. They are easily modified to include tags or reporter molecules of choice. ScFv expression is controlled by growth in a chemically defined auto-induction medium, which removed the necessity to monitor bacterial growth by optical density. All of the scFv formats produced from these vectors can be used in a variety of assays directly from either periplasmic or total cell lysates without the need for purification. One of the vectors, pSANG14-3F, which produces scFv-fused to alkaline phosphatase, offers a simple and convenient way of determining antibody fragment binding in a variety of assay systems without the need for antibody fragment purification and secondary antibody reagent addition. Furthermore, the bivalent scFv-AP has been shown to confer increased sensitivity over monomeric scFv in ELISA, western blot, and immunohistochemistry.

## Methods

### Reagents, scFv clones & bacterial strains

Unless otherwise specified, all restriction endonucleases and DNA modifying enzymes were obtained from New England BioLabs (Hitchin, Herts, UK) or Roche Applied Sciences (Lewes, UK). pMAL-c2x plasmid was obtained from New England BioLabs (Hitchin, Herts, UK). All electrophoresis and western blotting reagents were obtained from Invitrogen (Paisley, UK). All the other reagents were of analytical grade and purchased from Sigma-Aldrich (Poole, Dorset, UK). *E. coli *BL21 (DE3) and Origami 2™ (DE3) cells and the pET26b(+) plasmid were from Novagen (San Diego, CA). HER2 specific scFvs were kindly donated by Dr J. Marks (University of California, San Francisco). The other scFvs were generated in house to commercially available antigens purchased from R&D Systems (MN, USA), USBiologicals (MA, USA) and Progen (Heidelberg, Germany).

### Vector construction

All vectors were constructed using the vector pET26b(+) (Novagen) as the backbone. pSANG10 and pSANG11 vectors were created by digesting pET26b(+) at the single *Xho*I site and at the *Bgl*I sites closest to the T7 promoter (by partial digestion) and replacing the fragment with a double stranded oligonucleotides compatible with these sites, using four annealed oligonucleotides: CysBgSaS, CysBgSaA, SerBgSaS, and SerBgSaA (see Table 2 for all oligonucleotide sequences). This introduces *Nco*I, *Pst*I, *Not*I, *Hind*III restriction sites and a serine (pSANG10) or a cysteine (pSANG11) immediately after the *Not*I site. The resultant pSANG-10 vector sequence between the *Bgl*I and *Xho*I cloning sites is shown in Figure 1.

pSANG15, the leaderless equivalent of pSANG10, was created by replacing the *Xba*I*/Nco*I fragment of pSANG10 which contains the *pelB *leader sequence, with a short insert generated from two annealed oligonucleotides: Leadless and leadlesA. The maltose-binding protein (MBP) encoding gene from pMAL-c2x was inserted into pSANG10 at *Pst*I*/Hind*III sites. The MBP fragment was amplified using either PstMBP and MBPCysHind or PstMBP and MBPSerHind to include a *Not*I site at the 5' end of the fragment, and a cysteine (pSANG12) or a serine (pSANG13) residue and the hexa-histidine tag at the 3' end. The insertion of the MBP gene into the leaderless vector, pSANG15, to create pSANG16 (MBP-ser) and pSANG17 (MBP-cys) was achieved using the same strategy as above but using NcoMBP as the 5' primer instead of PstMBP.

The alkaline phosphatase encoding gene was cloned into *Pst*I*/Hind*III site of pSANG10 to create pSANG14. Prior to insertion, we had introduced a silent mutation at nucleotide 828 (T→C) to destroy an internal *Nco*I. This was achieved by PCR amplification and assembly of the alkaline phosphatase gene using the products of pstAlkP and mutAPantisense, and mutAPsense and alkPSerHind primers. The plasmid pEZZ707 [6] containing a mutant alkaline phosphatase gene with improved enzymatic activity were obtained from Dr B. Kay (Argonne National Laboratory, USA).

The tri-FLAG epitope tag (Figure 1a) was inserted into pSANG10, pSANG11, pSANG14 and pSANG15 at the *Hind*III site using two annealed oligonucleotides, H3flagA and H3flagS, to create pSANG10-3F, pSANG11-3F, pSANG14-3F and pSANG15-3F. Finally, pSANG18-3F (leaderless plus C-terminal cysteine) was constructed by cloning the *Nco*I/*Not*I, cysteine, hexa-histidine, tri-FLAG components from pSANG11-3F into the leaderless construct pSANG16. This was done by cloning an *Nco*I/*Pvu*I product of pSANG11-3F into the *Nco*I/*Pvu*I site of pSANG16 (*Pvu*I is present as a single site within the backbone of the original pET26 vector). All of the constructs were sequenced verified using the T7promoter2 and T7terminator primers.

### scFv expression and His-tagged protein purification

Antibody fragment expression was induced using a chemically defined protein expression induction medium [11]. A pre-culture was prepared in MDG media [25 mM Na_2_HPO_4_, 25 mM KH_2_PO_4_, 50 mM NH_4_Cl, 5 mM Na_2_SO_4_, 2 mM MgSO_4_, 10 μM Fe^+9^, 27.8 mM glucose, 18.8 mM aspartate] containing 50 μg/ml kanamycin. The MDG media was supplemented with 0.1 mg/ml of leucine for the Origami 2™ (DE3) strain. The pre-cultures were then incubated shaking overnight at 30°C. The inoculation of the induction cultures was normalised according to the optical density of the overnight pre-cultures. The auto-induction media; ZYM5052 [1% N-Z-amine AS, 0.5% yeast extract, 25 mM Na_2_HPO_4_, 25 mM KH_2_PO_4_, 50 mM NH_4_Cl, 5 mM Na_2_SO_4_, 2 mM MgSO_4_, 10 μM Fe^+9^, 54 mM glycerol, 2.8 mM glucose, 5.6 mM α-lactose]; was supplemented with 50 μg/ml kanamycin and the cultures were grown overnight at 30°C shaking. To prepare the total bacterial cell lysate, the cells were pelleted at 3000 × g for 10 min., the supernatant was discarded and replaced by 1/10th of the initial culture volume of BugBuster^® ^reagent containing 25 U/ml benzonase, 2 μl/ml protease inhibitor cocktail VII and 1.5 U/ml r-lysozyme. The cells were resuspended and lysed by agitation at room temperature for 10–20 min. and centrifuged at 16,000 × g for 20 min. to pellet the debris.

Where purified scFvs were used, these were generated from periplasmic extracts purified on Ni-NTA agarose (Qiagen, Crawley, UK). The periplasmic extracts were prepared by resuspending cells from the overnight induction cultures in 1/20th of the initial culture volume with TES [30 mM Tris-HCl pH 8.0; 1 mM EDTA; 20% sucrose (w/v)] containing 25 U/ml benzonase, 2 μl/ml protease inhibitor cocktail VII and 1.5 kU/ml r-lysozyme. After 10 min. incubation on ice, the cells were pelleted at 3,000 × g for 10 min. and the supernatant was decanted and kept on ice. The cell pellet was resupended in an equal volume of 5 mM MgSO_4 _supplemented with 25 U/ml benzonase, 2 μl/ml protease inhibitor cocktail VII and 1.5 kU/ml r-lysozyme and incubated on ice for 10 min.; after 10 min. centrifugation at 3,000 × g, the supernatants were pooled and a final clarifying spin at 16,000 × g for 20 min. was performed. The periplasmic extracts were also passed through a 0.45 μm filter before purification. The Ni-NTA agarose was equilibrated in 2 × PBS pH 8.0 plus 10 mM imidazole and 40 μl of slurry was added per millilitre of extract then mixed on a rotary mixer for 1 h at 4°C. The resin was then washed 5 times by centrifugation in 2x PBS pH 8.0 plus 20 mM imidazole. The his-tagged scFv were eluted in 1/10th of initial volume of protein extract in 50 mM Tris, 400 mM imidazole, 500 mM NaCl, pH 8.0. The samples were dialysed for 24 h at 4°C against PBS pH 8.0 plus 0.01% NaNO_3_, supplemented with 50 μM ZnSO_4 _and10 mM MgSO_4 _for scFv-AP expressed from pSANG14-3F.

ScFv expression was also performed by induction with 1 mM IPTG. Bacteria were grown in 2TY, 20% glucose, 50 μg/ml kanamycin for approximately 6 h to give an A_600 _value of 0.5–1.0. The cells were pelleted at 3000 × g for 10 min. and resuspended in an equal volume of 2TY, 50 μg/ml kanamycin, IPTG 1 mM and incubated overnight shaking at 30°C.

### ELISA

Detection of antigen binding by scFvs was conducted by coating the antigens in 96 well Maxisorp NUNC™ Immuno Plate at 5 μg/ml in phosphate buffered saline (PBS) or carbonate buffer (pH 9.6) according to the antigen used, 50 μl/well, overnight at 4°C. The wells were then blocked for 1 h at room temperature with 150 μl of 3% skimmed milk-phosphate buffered saline (M-PBS). After blocking, the wells were washed 3 times with PBS-0.1% Tween 20 (PBS-T) and 3 times with PBS. After scFv induction, dilutions of the total cell lysate or of scFvs purified from periplasmic extracts were prepared in M-PBS. Fifty microlitres of blocked scFv was then added to each well of the ELISA plate and incubated for 1 h at room temperature. The washing step was repeated as above and 50 μl/well of Eu-N1 labelled anti-FLAG M2 antibody (Sigma/Perkin Elmer, UK) was added at 0.6 ng/μl in M-PBS for 1 h at room temperature. After washing, the plates were incubated for 10 min. shaking at room temperature with 50 μl of DELFIA^® ^enhancing solution (Perkin Elmer, UK). The europium signal was read using time resolved fluorescence on a Fusion™ instrument (Perkin Elmer, UK). Each assay was performed in duplicate, with the mean signal value plotted. When analysing the effect of the fusion partner choice on yield for a range of scFvs, the ELISA data were then normalised to the results obtained for the standard monovalent scFv format (from pSANG10-3F), *i.e. *the ELISA score for the monovalent scFv format was given a value of 1.0 and the ELISA values for the other scFv formats determined as a fraction of this value. For direct detection of alkaline phosphatase, the scFv binding step was followed by the washes as described previously and supplemented with an extra wash with dH_2_O. Two hundred microlitres per well of SIGMA*FAST*™ p-Nitrophenyl Phosphate was added for 30 min. at room temperature, and the alkaline phosphatase reaction was read at 405 nm.

### SDS-PAGE and densitometry analysis

Proteins were separated by SDS-PAGE in a NUPAGE^® ^Novex Bis-Tris 4–12% electrophoresis gel. Samples were loaded at 2 μl per lane and one lane per gel of an scFv standard was run for quantification. The gel was then stained with SYPRO^® ^Red protein gel stain following manufacturer's instructions (Invitrogen Paisley, UK). The gel was visualised on a Typhoon 9410 scanner, the scanned gels were analysed using ImageQuant TL software (Amersham Biosciences, UK).

### Western blot

Recombinant human desmin (Progen, Heidelberg, Germany) and mouse skeletal muscle tissue lysate (Abcam plc, Cambridge, UK), were diluted in PBS to a final volume of 10 μl to which was added 4 μl of loading buffer (containing NUPAGE^® ^LDS sample buffer plus Invitrogen reducing agent). The proteins were separated under reducing conditions in NUPAGE^® ^Novex Bis-Tris 4–12%. The proteins were transferred to an Invitrolon™ PVDF membrane using an electroblotting apparatus, and incubated for 1 h at room temperature in M-PBS plus 0.1% Tween 20 (M-PBS-T). The membranes were washed 3 times for 5 min. each in PBS plus 0.1% Tween 20 (PBS-T) and incubated with 0.4 μg/ml of anti-histidine tag mouse mAb (Merck Biosciences Ltd., UK) in M-PBS-T for 1 h at room temperature on an orbital shaker. After 3 washes of 5 min. each in PBS-T, the membranes were incubated as described above but in the dark with 0.4 μg/ml of goat anti-mouse Cy5 labelled antibody (Amersham Biosciences, UK). Finally, the membrane was washed 3 times for 5 min. each in PBS-T, once in water for 5 min. and then air dried. Where anti-FLAG and ECL detection was required, the membranes were incubated with 0.22 μg/ml of monoclonal Anti-FLAG^® ^M2 peroxidase (HRP) (Sigma-Aldrich, Poole, UK) in M-PBS-T for 1 h at room temperature on an orbital shaker, and ECL western blotting substrate added according to the supplier's instructions (Pierce, Cramlington, UK). The membranes were scanned on the Typhoon scanner and analysed using ImageQuant software. For alkaline phosphatase detection in western blot, scFv or scFv-AP, expressed from pSANG10-3F or pSANG14-3F respectively, purified or in diluted crude bacterial cell lysates were used as the primary antibody as described above. For indirect detection, 1.1 μg/ml of mouse monoclonal anti-FLAG^® ^M2-Alkaline Phosphatase was used as secondary antibody. The detection was performed using SIGMA*FAST *™ NBT/BCIP following the manufacturer's instructions (Sigma-Aldrich, Poole, UK).

### Immunohistochemistry

All immunohistochemistry was performed on an automated immunostainer using supplied reagents unless stated otherwise (Ventana, Tucson, AZ). For peroxidase detection, frozen sections were air-dried for 30 min. prior to fixation in fresh acetone for 15 min. (BDH). Endogenous peroxidase was blocked for 20 min. in 0.003% H_2_0_2_/100% methanol and then transferred to reaction buffer before the run commenced. The sections were incubated with pSANG10-3F or pSANG14-3F-derived scFv for 12 h at room temperature, before addition of biotinylated anti-FLAG for 60 min. Detection was effected through tyramine signal amplification based on tyramine-DNP. Detection of the deposited biotinylated tyramine and visualised via a streptavidin-peroxidase conjugate with 3, 3-diaminobenzidine tetrahydrochloride (DAB) substrate and copper enhancement giving a brown coloured end product All slides were counterstained in haematoxylin, before manual dehydration through alcohols, clearing in xylene and mounting in DPX. Endogenous biotin was blocked using an endogenous biotin blocking kit. Non-specific protein blocks were present in the supplied buffers.

For alkaline phosphatase detection, frozen sections were air-dried and acetone fixed as before, however endogenous peroxidase blocking was replaced with 10 min. incubation in dual endogenous enzyme block (DAKO UK Ltd) to block endogenous alkaline phosphatase activity. pSANG14-3F-generated scFv were incubated for only 1 h at room temperature, followed by either direct detection of the bacterial alkaline phosphatase or incubation for 60 min. with anti-FLAG AP. ScFv binding was revealed by 60 min. incubation with NitroBlue Tetrazolium/Bromo-chloro Indoyl Phosphate (NBT/BCIP) substrate. The link streptavidin-AP within the kit was not used. Slides were counterstained with Nuclear Fast Red (Vector Labs, UK) and mounted in Immu-mount (Thermo Shandon, UK).

### Quantitative image analysis

(1) A CCD colour camera (Megaplus ES4.0/E, mounted on Olympus BX61) was used to capture the tissue samples, which resulted in a microscopic image (3,800 × 3,800 pixels, 24BPP) in the form of red-green-blue (RGB) model. (2) An image pre-processing is performed, where the image noise and uneven illumination background are removed by the Gaussian smoothing filter and top-hat filter respectively [20]. (3) Use of RGB model is problematic because these three intensities reflect both the absorption characteristics of the stains used and the amount to which those stains are present. An alternative to the extraction of the chromatic information from the image data is the hue-saturation-intensity (HSI) model. For the HSI model, classification of stains is based on two dimensions, hue (dominant wavelength) and saturation (purity of the colour). A region of interest stained with a given colour can, in principle, be identified by thresholding the three HSI channels. At the same time, the intensity value of stains/expression level can be measured via the corresponding saturation image. (4) For the fourth stage of the algorithm, the Canny edge detector [21] and properties of tissue morphology are used to distinguish between the tissue and the slide background. This allows the expression objects/stains to be characterized and unionized over structures of interesting tissue in the later step. Finally, the expression scores are expressed as the labelled stains signal intensity divided by the presented tissue area.

## Authors' contributions

CM participated in the vector construction, scFv expression analysis, scFv utility studies, and manuscript preparation. GRD participated in vector construction, scFv expression analysis, and scFv utility studies. JM carried out the IHC studies. KJV participated in the vector construction. JW designed the IHC image analysis algorithm and performed the analysis of the image data. DJS participated in the experimental design, co-ordination, and drafted the manuscript. JMc conceived the design of the expression vectors, contributed to experimental design, co-ordination, and helped to draft the manuscript. All authors read and approved the final manuscript.

## Figures and Tables

**Table 1:** A summary of the pSANG expression vector series, their scFv properties, and *E. coli *strains used.

**Vector Name**	**pSANG10-3F**	**pSANG11-3F**	**pSANG12**	**pSANG13**	**pSANG14-3F**	**pSANG15-3F**	**pSANG16**	**pSANG17**	**pSANG18-3F**
**Valency**	Monovalent	Bivalent	Monovalent	Bivalent	Bivalent	Monovalent	Monovalent	Bivalent	Monovalent
**Epitope Tag(s)**	6xHis Tri-Flag	6xHis Tri-Flag	6xHis	6xHis	6xHis Tri-Flag	6xHis Tri-Flag	6xHis	6xHis	6xHis Tri-Flag
**Site of scFv accumulation**	Periplasm	Periplasm	Periplasm	Periplasm	Periplasm	Cytoplasm	Cytoplasm	Cytoplasm	Cytoplasm
**Additional modifications**	None	C-terminal Cysteine	MBP	MBP, C-terminal Cysteine	Bacterial Alkaline Phosphatase	Leaderless	Leaderless, MBP	Leaderless, MBP, C-terminal Cysteine	Leaderless, C-terminal Cysteine
**Expression Host tested**	BL21(DE3) Origami 2	BL21(DE3)	BL21(DE3)	BL21(DE3)	BL21(DE3) Origami 2	Origami 2	Origami 2	Origami 2	Origami 2
**Protein expressed**	scFv	scFv-cys	scFv-MBP	scFv-MBP-cys	scFv-AP	scFv	scFv-MBP	scFv-MBP-cys	scFv-cys

**Table 2:** A summary of the oligonucleotides used to generate and sequence confirm the pSANG expression vectors.

**Primer Designation**	**Primer sequence (5'-3')**
CysBgSaS	5'-3cggccatggcccaggtgcagctgcaggcggccgcatgcgcacatcatcatcaccatcacaagctttaataag-3'
CysBgSaA	5'-3tcgacttattaaagcttgtgatggtgatgatgatgtgcgcatgcggccgcctgcagctgcacctgggccatggccggct-3'
SerBgSaS	5'-3cggccatggcccaggtgcagctgcaggcggccgcatccgcacatcatcatcaccatcacaagctttaataag-3'
SerBgSaA	5'-3tcgacttattaaagcttgtgatggtgatgatgatgtgcggatgcggccgcctgcagctgcacctgggccatggccggct-3'
Leadless	5'-ctagaaataattttgtttaactttaagaaggagatatacc-3'
leadlesA	5'-catgggtatatctccttcttaaagttaaacaaaattattt-3'
PstMBP	5'-nnnnnnctgcaggcggccgcaaaaatcgaagaaggtaaactggtaatc-3'
MBPCysHind	5'-nnnnnnaagcttgtgatggtgatgatgatgtgcacaagtctgcgcgtctttcagggcttcatcgac-3'
MBPSerHind	5'-nnnnnnaagcttgtgatggtgatgatgatgtgcagaagtctgcgcgtctttcagggcttcatcgac-3'
NcoMBP	5'-gcccagccggccatggcccaggtgcagctgcaggcggccgcaaaaatcgaagaaggtaaactggtaatc-3'
mut AP sense	5'-gcaacgtaccacggcaatatcgat-3'
mut AP antisense	5'-atcgatattgccgtggtacgttgc-3'
pstAlkP	5'-nnnnnnctgcaggcggccgcaaccccggaaatgcctgttctggaaaaccgg-3'
alkPSerHind	5'-nnnnnnaagcttgtgatggtgatgatgatgtgcagacggtactttcagccccagagcggctttcat-3'
H3flagS	5'-3agctggactacaaagaccatgacggtgattataaagatcatgacatcgattacaaggatgacgatgacaagtaataaa-3'
H3flagA	5'-3agcttttattacttgtcatcgtcatccttgtaatcgatgtcatgatctttataatcaccgtcatggtctttgtagtcc-3'
T7promoter2	5'-gatcgagatctcgatcccgcga-3'
T7terminator	5'-gctagttattgctcagcg-3'
